# Archaeal dominated ammonia-oxidizing communities in Icelandic grassland soils are moderately affected by long-term N fertilization and geothermal heating

**DOI:** 10.3389/fmicb.2012.00352

**Published:** 2012-10-04

**Authors:** Anne Daebeler, Guy C. J. Abell, Paul L. E. Bodelier, Levente Bodrossy, Dion M. F. Frampton, Mariet M. Hefting, Hendrikus J. Laanbroek

**Affiliations:** ^1^Department of Microbial Ecology, Netherlands Institute of Ecology (NIOO-KNAW)Wageningen, Netherlands; ^2^Institute of Environmental Biology, University of UtrechtUtrecht, Netherlands; ^3^CSIRO, Marine and Atmospheric Research and Wealth from OceansHobart, TA, Australia

**Keywords:** ammonia-oxidizing archaea, ammonia-oxidizing bacteria, fertilization, temperature, *amoA*, niche formation, grassland soil

## Abstract

The contribution of ammonia-oxidizing bacteria and archaea (AOB and AOA, respectively) to the net oxidation of ammonia varies greatly between terrestrial environments. To better understand, predict and possibly manage terrestrial nitrogen turnover, we need to develop a conceptual understanding of ammonia oxidation as a function of environmental conditions including the ecophysiology of associated organisms. We examined the discrete and combined effects of mineral nitrogen deposition and geothermal heating on ammonia-oxidizing communities by sampling soils from a long-term fertilization site along a temperature gradient in Icelandic grasslands. Microarray, clone library and quantitative PCR analyses of the ammonia monooxygenase subunit A (*amoA*) gene accompanied by physico-chemical measurements of the soil properties were conducted. In contrast to most other terrestrial environments, the ammonia-oxidizing communities consisted almost exclusively of archaea. Their bacterial counterparts proved to be undetectable by quantitative polymerase chain reaction suggesting AOB are only of minor relevance for ammonia oxidation in these soils. Our results show that fertilization and local, geothermal warming affected detectable ammonia-oxidizing communities, but not soil chemistry: only a subset of the detected AOA phylotypes was present in higher temperature soils and AOA abundance was increased in the fertilized soils, while soil physio-chemical properties remained unchanged. Differences in distribution and structure of AOA communities were best explained by soil pH and clay content irrespective of temperature or fertilizer treatment in these grassland soils, suggesting that these factors have a greater potential for ecological niche-differentiation of AOA in soil than temperature and N fertilization.

## Introduction

Ammonia-oxidizing archaea (AOA) are among the most abundant archaeal organisms known on earth with an almost ubiquitous distribution. In soils they most often co-occur with ammonia-oxidizing bacteria (AOB), a fact which raises questions about the ecophysiology and ecological importance of AOA in relation to AOB. Evidence is building, that the ecological significance of AOA vs. AOB varies depending on soil environmental conditions, which set the dimensions of niche segregation for AOA and AOB. Both ammonia-oxidizing communities may be influenced by a number of soil environmental factors including concentration of NH^+^_4_, organic carbon, oxygen concentration, sulphide, phosphate, temperature, pH, ammonium source, and pore water redox condition (Erguder et al., 2009; Schleper and Nicol, 2010). Several studies demonstrated enhanced AOB abundances and community shifts with a concomitant increase in nitrification rates after the addition of organic and/or mineral fertilizers to various soils whereas the AOA remained unaffected or even decreased in number (Horz et al., 2004; Enwall et al., 2007; Di et al., 2010; Fan et al., 2011; Jung et al., 2011; Shen et al., 2011; Verhamme et al., 2011; Wertz et al., 2012). In contrast to these findings, a stimulation of transcriptional activity of the archaeal *amoA* gene after incubation of grassland soil with addition of ammonia has been reported by Treusch et al. (2005). Moreover, an increasing mass of contrasting results from laboratory and field experiments (e.g., He et al., 2007 and Kelly et al., 2011 vs. Hallin et al., 2009 and Lamb et al., 2011; Tourna et al., 2008 vs. Fierer et al., 2009 and Avrahami et al., 2011) points to the possibility that the relevance of substrate concentration and temperature for niche segregation of AOA and AOB in soil might not be independent, but that the effect of these factors varies in concert with other soil environmental properties. Indeed, Jung et al. (2011) found that mineral N-fertilization could balance a separately observed negative effect of warming on the size of ammonia-oxidizing communities.

In this study we examined the discrete and combined effects of mineral nitrogen deposition and temperature on ammonia-oxidizing communities in the context of several soil physico-chemical properties by sampling soils from ambient temperature as well as geothermally heated grassland sites in Iceland. These sampling sites, covering a natural temperature gradient, have undergone a long-term fertilization. We hypothesized that temperature would primarily affect AOA community structures without affecting its size, while fertilization would diminish AOA and favor AOB populations. Our set up additionally allowed studying interactive effects of temperature and fertilization and we expected the fertilized, geothermally heated sites to harbor less diverse, AOB dominated communities. Microarray, clone library and quantitative PCR analyses of the ammonia monooxygenase subunit A (*amoA*) gene accompanied by measurements of various soil properties were conducted.

## Materials and methods

### Sample collection and physico-chemical analyses

**Figure 1:**
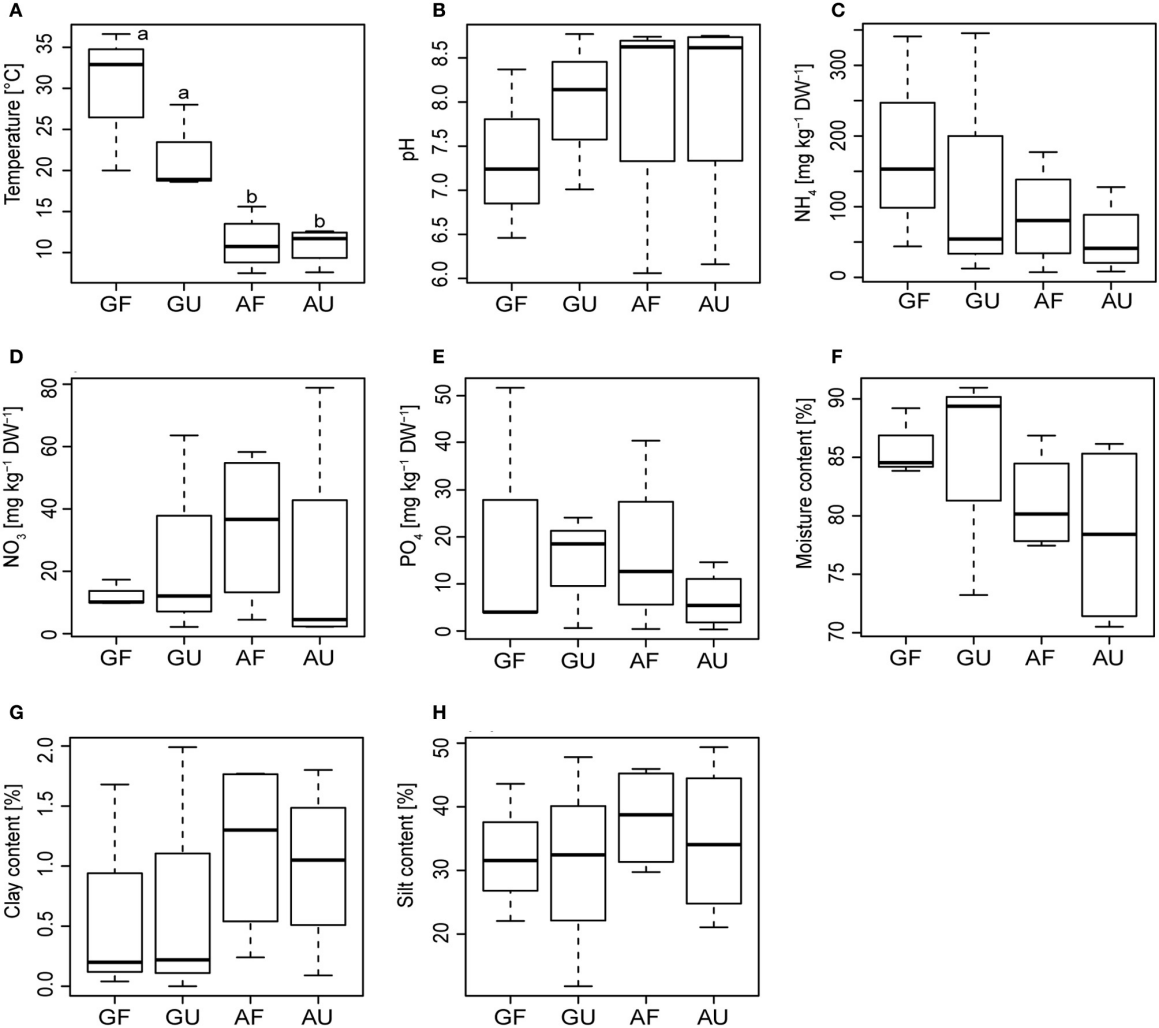
**Boxplots (*n* = 3–4 samples) depicting the soil physico-chemical properties of the four soil groups.** The median is indicated at the midpoint, the hinges indicate the upper and lower quantiles, and the lines represent the spread. Small cap letters in **(A)** indicate significant differences based on Tukey's HSD test (*p* < 0.05), no significant differences were found in **(B–H)**. GF = geothermally heated and fertilized; GU = geothermally heated and unfertilized; AF = ambient temperature and fertilized; AU = ambient temperature and unfertilized.

Grassland soil samples were taken in Grændalur valley (64° 1′ 7″ N, 21° 11′ 20″ W), Iceland, in May 2009 before the annual fertilization of the experimental plots took place. Soils are Histic Andisols with two tephra layers in the top 30 cm. At time of sampling the mean air temperature ranged from 3.6°C to 9.4°C, but because of geothermal activity in the valley these grassland soils had various temperatures ranging from 8°C to 36.5°C (see Figure 1A). Soil temperatures have been monitored since the establishment of the experimental field sites in 2005 and only sites that have since then been stable in their geothermal or non-geothermal influence were selected for this study. Fertilization with chemical, slow release urea fertilizer (Agroblen Base 35+00+00, Everris International B.V., The Netherlands) was applied in mid-May of every year by equally sprinkling granular solids onto the soil. Soil cores (top 15 cm) were collected from four ambient temperature and three geothermally influenced sites, each of which is comprised of a subplot that has been fertilized annually since May 2005 (10 g N·m^−2^ a^−1^) and an unfertilized control subplot. From each subplot three replicate cores were collected and mixed forming a composite sample to account for spatial heterogeneity in each subplot. Only the upper 10 cm of the cores were used for analyzes. Each sample was placed in a sterile plastic bag and kept at 4°C during transport to the laboratory. After removal of roots and mixing, the samples were stored at −20°C for DNA extraction and at 4°C for analysis of soil physico-chemical properties. The different soil samples are further designated as G and A for geothermally influenced and ambient temperature respectively and U and F for unfertilized and fertilized, resulting in four soil groups: GU, GF, AU, and AF.

Soil moisture content was determined by measuring mass loss percentage after 48 h of drying at 70°C. For chemical analysis 100 ml of demineralized water was added to 15 g of fresh soil. The slurries were shaken for 1 h and centrifuged for 4 min at 4000 rpm. The supernatant was filtered (Whatman GF/C) and stored at −20°C. The samples were analyzed colorimetrically for NO^−^_3_, NH^+^_4_, and PO^−^_4_ on a continuous flow auto-analyzer (SA-40, Skalar Analytical BV, The Netherlands). The demi-water extracts were also used for soil pH measurements. Soil clay and silt content were analyzed from freeze-dried subsamples by a Mastersizer (Malvern, model APA 2000, serial number 34403/139).

### DNA extraction from soil, amplification of AOA and AOB amoA, cloning, and microarray

Nucleic acids were extracted from ~0.5 g of soil according to Lueders et al. (2004). Polymerase chain reaction (PCR) thermal profiles, reaction mixtures and primers used for amplification of the archaeal *amoA* gene can be found in Table A1.

Several PCR assays and primer pairs were tested to amplify DNA of ammonia-oxidizing bacteria (see Table A2). Two nested approaches using the primer pairs βAOBf/βAOBr (McCaig et al., 1994) and CTO189f/CTO654r (Kowalchuk et al., 1997) and A189/amoa2-R (Holmes et al., 1995; Rotthauwe et al., 1997) and amoA1-F/amoa2-R (Rotthauwe et al., 1997), targeting the 16S rDNA and the *amoA* gene, respectively, delivered products of the expected size, *albeit* in concentrations too low for cloning or microarray analysis.

Archaeal *amoA* gene copy numbers were quantified using the Rotor-Gene 3000 real-time PCR system (Corbett Research) with ABsolute Q-PCR SYBRgreen mix (AbGene). All quantitative PCR data were obtained from samples and non-template controls subjected to duplicate independent amplification. Description of the 25-μL reaction mixture, thermal profile and primers used can be found in Table A1. A standard curve for quantification of AOA *amoA* was generated from 10-fold serial dilutions (10^2^−10^8^ copies μL−1) of a purified SP6/T7-PCR product from clone 29C_47 (accession number JQ4 04089, this study) containing an archaeal *amoA* fragment. The detection limit of the AOA *amoA* qPCR assay was 7.27 × 10^3^ copies/g of dry soil, corresponding to 4.2 copies per reaction.

To quantify bacterial *amoA* with various primer pairs, cycling conditions, sample DNA concentrations and two SYBR-Premixes were tested (see Table A2), but no amplification could be achieved.

As the qPCR results indicated a very strong dominance of AOA over AOB, we focused on AOA for analysis of community structures. A microarray platform targeting the *amoA* genes of AOA was used as described in PLoS1 (under revision). Processing of the data included standardization according to an internal standard on the array, elimination of false positive as well as redundant signals and determination of representative sequences for true, positive signals by determining representative sequences for the phylogenetic clade targeted by the probe. A list with probe signals included in the analysis, as well their representative sequences, is provided in Table A3.

Cloning of archaeal *amoA* gene fragments was conducted with pooled triplicate PCR reaction products. Samples were purified using QIAquick PCR Purification Kit (Qiagen), cloned by ligation into pGEM-T vector plasmids (Promega) and transformation of *Escherichia coli* competent cells JM109 (Promega) according to the manufactures' instructions. In total 14 clone libraries were constructed, containing on average 50 clones per library.

### Sequencing and phylogenetic analysis

The bi-directional sequences of positive clones were assembled with Sequencher 4.2 (Gene Codes Corporation) and compared with sequences available in the GenBank database using the BLAST network service to determine the approximate phylogenetic affiliations. Prior to analysis all sequences containing stop codons within the reading frame were discarded. Alignment and phylogenetic analysis of *amoA* sequences from all 14 clone libraries together with representative sequences for all true positive signals of the microarray was done in ARB (Ludwig et al., 2004). Calculation of operational taxonomic units (OTU) with average neighbor algorithm at 15% nucleotide sequence divergence level, as well as determination of representative clones for the OTUs found by clone library analysis, was carried out using the software mothur 1.19.3 (Schloss et al., 2009). All representative sequences of the defined OTUs were checked for chimeras by blasting both halves of the sequences separately. The relative abundances of the OTUs were then used as community profiles to evaluate the similarity between the four soil groups. The archaeal *amoA* sequences generated in this study have been deposited in DDBJ/EMBL/GenBank nucleotide databases under accession numbers JQ403649–JQ404406.

### Statistical analysis

Soil property data and AOA community data was standardized and/or transformed by Box-Cox power transformation if necessary. To test for significant differences regarding measured soil physico-chemical properties, as well OTU richness, diversity and *amoA* gene copy number between the four soil groups, Tukey's HSD test was performed. Spearman rank (ρ) correlations were run to investigate the relationship between soil properties and the copy number of archaeal *amoA* genes, richness and Shannon diversity of the AOA communities as well as relative abundance of single phylotypes. Standardized Mantel (r) correlations (based on Pearson product-moment correlation coefficient, 999 permutations) were performed to test for relationships of total AOA community structure from the four soil groups with soil properties. All correlation results were subjected to Benjamini-Hochberg correction for multiple comparisons to protect from Type I errors. Analysis by two-way ANOVA was performed to test for interactive effects of fertilization and geothermal heating on OTU richness, OTU diversity as indicated by Shannon's H' index of diversity and *amoA* gene copy numbers.

Dissimilarity matrices of soil properties were calculated using Euclidian distances, while AOA community structure dissimilarity was calculated using Bray-Curtis' distances. The differences in relative abundance of OTUs between plots were analyzed by non-metric multidimensional scaling (NMS). The NMS analysis was conducted using function metaMDS which combines all recommendations for NMS analysis (Minchin, 1987). Biota-environment (BioEnv) analysis with the community dissimilarity matrix and the soil properties dissimilarity matrix was conducted to determine the best subset of measured soil properties for constrained correspondence analysis (CCA). Significance of the constraints applied to the CCA analysis was tested by permutation analysis. Soil properties and relative abundances of single OTUs were incorporated into the analysis through the usage of tri-plot ordination, where the variables were combined in a secondary matrix and plotted as linear vector fits against the community composition ordination. The goodness of each fit was tested by squared correlation coefficient (*R*^2^) tests and only significant fits were plotted into the ordination. All statistical analyzes were run in *R* ver. 3.12.2 (R Development Core Team., 2011). For multivariate analysis the vegan package ver. 2.1–0 (Oksanen et al., 2010) was used.

## Results

### Soil physico-chemical analysis

On average the geothermally heated soils (GU & GF) had a higher temperature, higher moisture, and lower clay content as well as a lower pH (Figures 1A,F,G,B) than the colder, ambient temperature soils (AU & AF), but temperature was the only significantly different variable (Figure 1A). None of the analyzed soil properties differed significantly between the fertilized (GF & AF) and the unfertilized soils (GU & AU). However, there was an indication of higher NH^+^_4_ content in the fertilized soils, although because of considerable variation within the groups this difference was insignificant (Figure 1C).

Contents of NO^−^_3_ were found to be correlated with pH and PO^−^_4_ contents (*p* < 0.05; ρ = 0.72 and *p* < 0.05; ρ = 0.67, respectively). Additionally, silt and clay content of the soils were correlated (*p* < 0.01, ρ = 0.78) and were in turn both negatively correlated with soil moisture content (both *p* < 0.005; ρ = −0.84 and −0.86).

### Abundance of AOA and AOB

**Figure 2:**
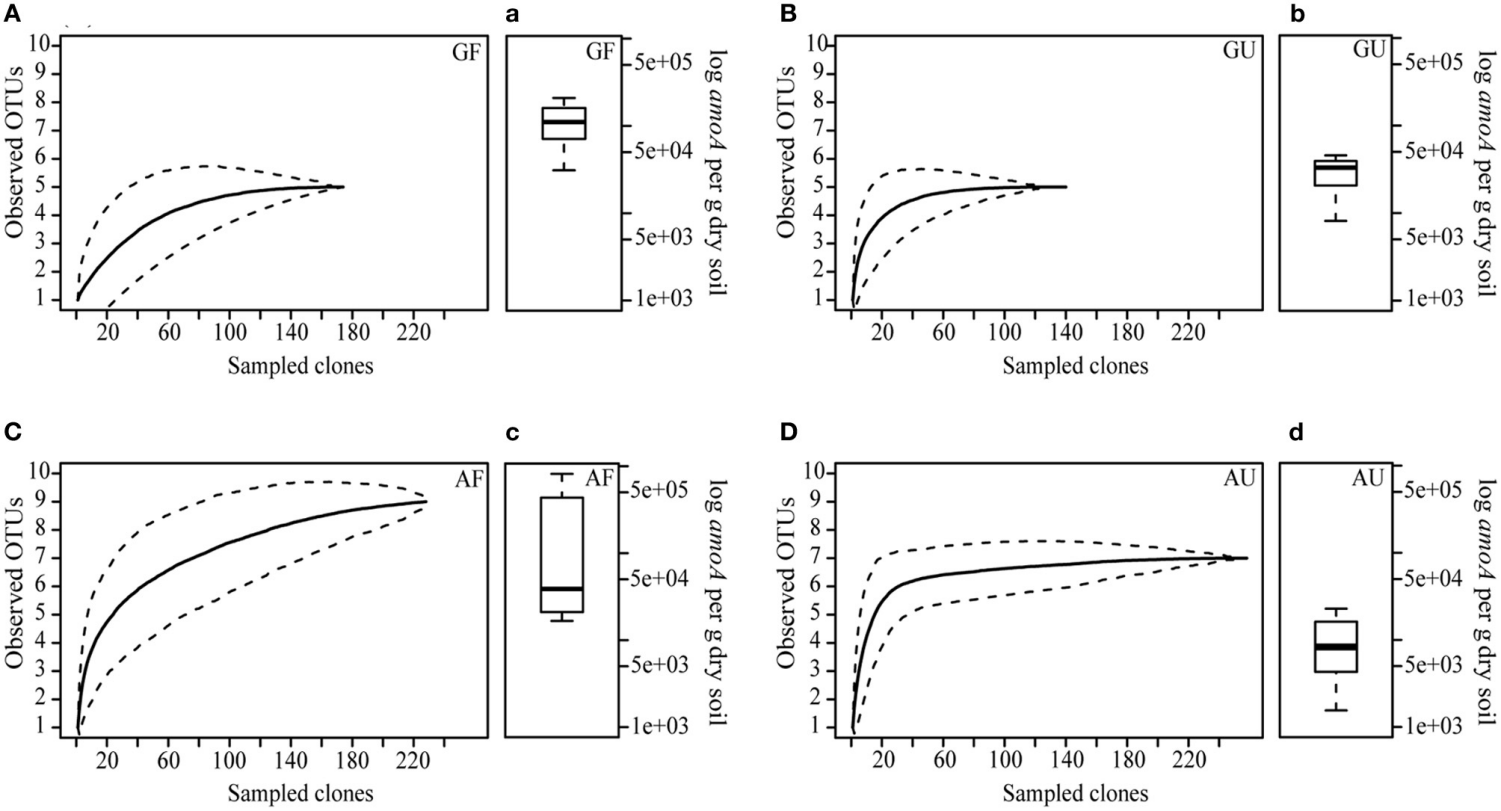
**Rarefaction curves for archaeal *amoA* clone libraries (A–D) as well as boxplots of archaeal *amoA* gene abundances (a–d) from the four different soil groups (*n* = 3 or 4). (A–D)** Shown are average numbers of observed OTUs defined at 15% nucleotide sequence dissimilarity (solid line) as well as the lower and upper 95% confidence intervals (dotted lines). OTUs were defined at nucleotide dissimilarity level of 15%. **(a–d)** depicted at the midpoint of each boxplot is the median; the hinges indicate the upper and lower quantiles and the lines represent the spread. GF, geothermally heated and fertilized soil; GU, geothermally heated and unfertilized soil; AF, ambient temperature and fertilized soil; AU, ambient temperature and unfertilized soil.

To quantify the abundance of the ammonia-oxidizing communities present in the four soil groups, qPCR targeting the *amoA* gene of AOA and AOB was performed; however, only archaeal *amoA* could be amplified. Abundance of archaeal *amoA* genes detected in the soils ranged from 1.55·10^3^ to 8.15·10^5^ copies per gram of dry soil, with highest numbers found in the AF soils (Figures 2a–d). Archaeal *amoA* abundance was lower in the GU and AU soils compared to the respective fertilized soils, however, this difference was not significant (ANOVA, *p* = 0.07), likely due to considerable variation within the soil groups.

Despite various efforts we were unable to detect bacterial *amoA* by quantitative PCR in the analyzed soils. Based on the 10-fold standard deviation around the average intensity of background fluorescence from non-template controls we estimated the detection limit to be 4.99 × 10^3^ copies/g of dry soil, corresponding to 9.1 copies in the reaction mixtures. We performed controls to estimate possible inhibition of PCR performance by co-extracted compounds in soil DNA by spiking standard samples with 1–3 μl of soil DNA sample. There was no detectable difference in the quantified bacterial *amoA* genes between standards with and without soil DNA addition and we therefore concluded that soil PCR inhibitors did not interfere with the quantitative PCR assay. We furthermore failed to amplify bacterial *amoA* genes from soil, which we incubated shaking at 180 rpm in a 1:1 volume slurry with 10 mM NH^+^_4_ AOB medium for 4 days. However, amplification of AOB by two nested PCR approaches, one targeting the 16S rDNA and the other targeting the *amoA* gene, yielded a product of the expected size. This led us to conclude that AOB inhabited the soils, *albeit* in numbers too low for detection by quantitative PCR based on the *amoA* gene.

### AOA community structure

**Figure A1:**
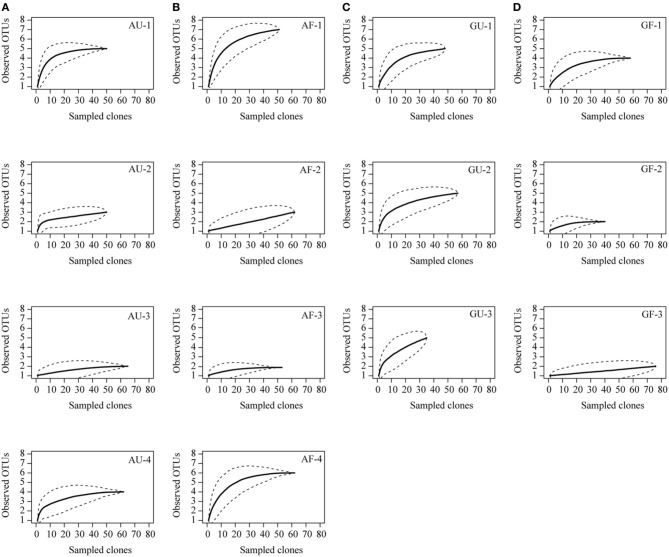
**Rarefaction curves of archaeal amoA genes retrieved from ambient temperature and unfertilized soils (A), ambient temperature fertilized soils (B), geothermally heated unfertilized soils (C) and geothermally heated fertilized soils (D).** Shown are estimated numbers of OTUs(solid line) as well as the lower and upper 95% confidence intervals(dotted lines). OTUs were defined at nucleotide dissimlarity level of 15%. GF = geothermally heated fertlizied soil; GU = geothermally heated unfertilized soil; AF = ambient temperature fertilized soil; AU = ambient temperature unfertilized soil.

Clone libraries of archaeal *amoA* genes amplified from the four soil groups were constructed to study the community structure of AOA. The rarefaction curve analysis of the respective clone libraries revealed that ambient temperature soils harbored a higher richness of OTUs (defined at 15% nucleotide dissimilarity) with highest observed richness in AF soils (Figures 2C,D). Only five OTUs were found in both of the geothermally heated soils GF and GU, respectively (Figures 2A,B). Except in the case of the AF soil, the rarefaction analysis also showed that the size of the clone libraries was sufficient to account for the diversity of the whole AOA communities inhabiting the analyzed soil. Two out of four rarefaction curves from ambient temperature soil clone libraries indicated increased OTU richness with fertilization as opposed to a trend of decreasing OTU richness with fertilization that was found in all geothermally heated soils (Figure A1).

**Figure 3:**
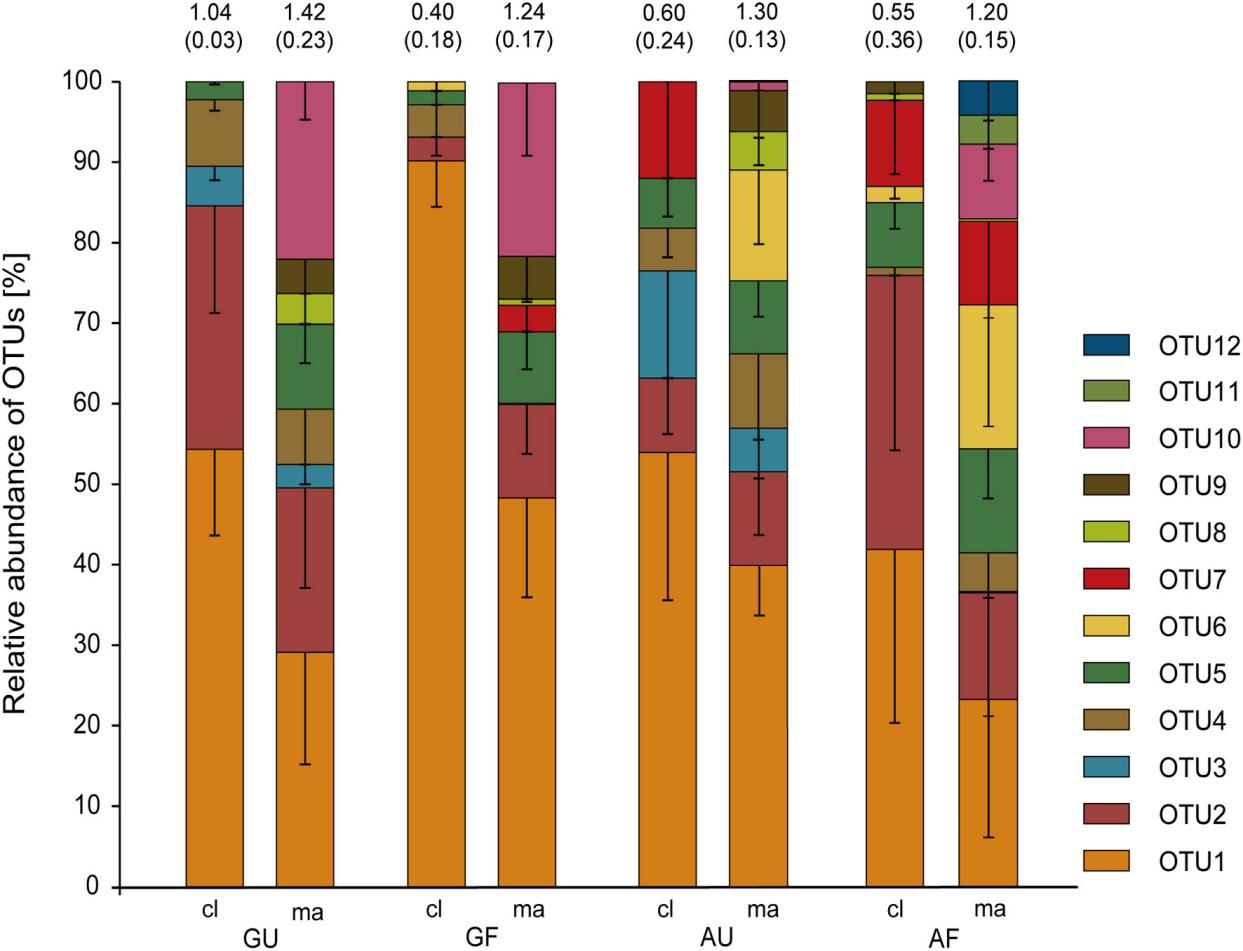
**Relative abundance of AOA *amoA* OTUs defined at 15% nucleotide sequence dissimilarity as detected by clone library analysis and microarrays.** Error bars denote standard errors (*n* = 3 or 4). Numbers above the bars are Shannon's index of diversity ± standard error in brackets. Cl, clone library; ma, microarray; GF, geothermally heated and fertilized soil; GU, geothermally heated and unfertilized soil; AF, ambient temperature and fertilized soil; AU, ambient temperature and unfertilized soil.

To further study the structure of AOA communities, we utilized an *amoA* microarray, designed to target the archaeal *amoA* sequences generated in this study. Microarrays and sequence analysis of clone libraries revealed similar AOA community compositions, but the microarray method had a higher sensitivity and detected three OTUs not detected by clone library analysis (Figure 3). OTU 1 clearly dominated the communities of all soils. OTUs 10, 11, and 12 were only detected using the microarray. Interestingly, OTU 3 was exclusively found in the unfertilized soils by both methods of community analysis. Figure 3 furthermore shows that communities of GU and GF soils were comprised of a subset of the OTUs found in the ambient temperature soils according to clone library and microarray analysis. Shannon's H' index of diversity of the AOA communities determined for both community analysis methods was lower for the fertilized compared to the unfertilized soils on average (Figure 3).

**Figure 4:**
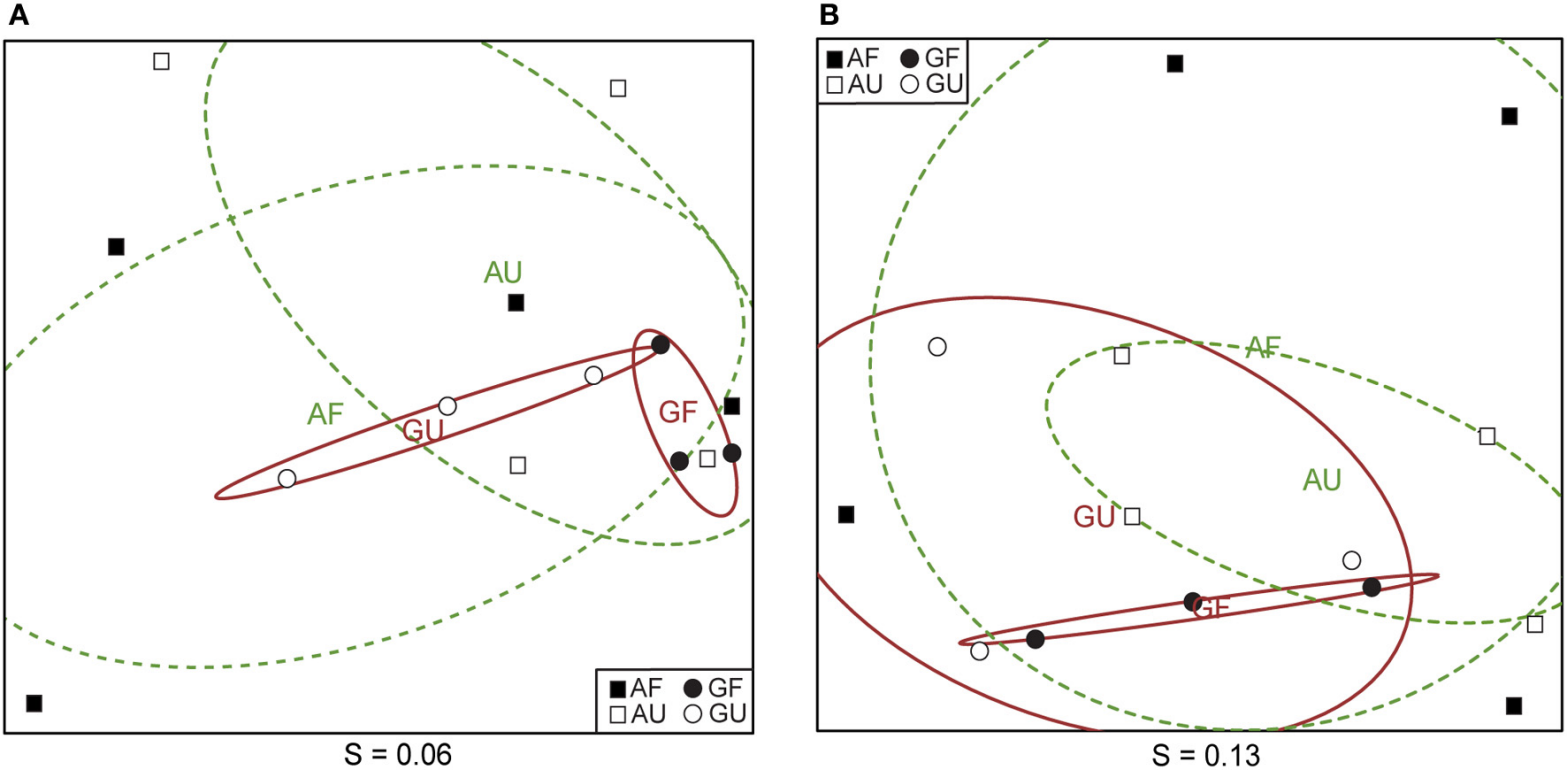
**Non-metric multidimensional scaling analysis of AOA communities as determined by clone library analysis (A) and microarrays (B) based on Bray-Curtis' distances of relative abundances of OTUs defined at 15% nucleotide sequence dissimilarity.** Confidence ellipses (95%) surround the centers of AOA communities derived from geothermally heated (solid red lines, GF and GU) and ambient temperature soils (dashed green lines, AF and AU). GF, geothermally heated and fertilized soil; GU, geothermally heated and unfertilized soil; AF, ambient temperature and fertilized soil; AU, ambient temperature and unfertilized soil. Both MDS routines applied by the metaMDS command were isoMDS, which utilizes PCO analysis as starting configuration. Stress values (S) are given below the figures, *r*^2^ = 0.996 and 0.986 for **(A)** and **(B)**, respectively.

Community composition analysis by NMS (Figure 4) gave similar separation for both methods of community analysis, pointing to insignificant differences in community structure between the four soil groups. Variation within the communities from the two geothermally heated soils was generally lower than in the ones from ambient temperature soils, where the 95% confidence intervals were substantially larger (Figure 4).

**Figure 5:**
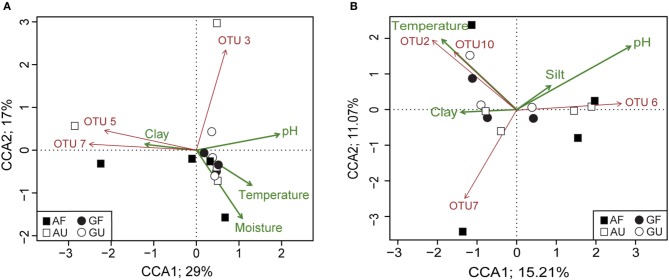
**Biplots of constrained correspondence analysis (CCA) of AOA communities based on relative abundances of OTUs defined 15% nucleotide sequence dissimilarity. (A)** AOA communities as determined by clone library analysis. Four constraints were applied: pH, moisture content, clay content, and temperature (*p* < 0.05); the CCA explains about 57% of overall variation, with CCA1 being the most important axis. **(B)** AOA communities as determined by microarray analysis. Four constraints were applied: pH, temperature, clay, and silt content (*p* < 0.05); the CCA explains about 39% of overall variation, with CCA1 being the most important axis. Green arrows indicate the direction in which constraints correlate with the ordination axes; red arrows indicate the direction in which significantly fitted OTUs (*p* < 0.05) correlate with the ordination.

BioEnv analysis selected the soil properties pH, moisture, and clay content as the subset of variables best correlating with the dissimilarity matrix of clone library OTUs, whilst the best correlating subset of soil properties for the microarray OTU dissimilarity matrix comprised pH, moisture, clay, and silt contents. These variables therefore have a stronger potential to explain community dissimilarities than the other measured soil properties. Based on these results, we conducted CCA for the clone library as well as the microarray derived community data followed by permutation analysis. In the case of the CCA with the clone library based data we found the highest explanatory power and significance for the soil properties pH, clay, and moisture content, as well as temperature as constraining variables (CCA; *p* < 0.05). The four applied constraints explained 57% of overall observed variation between the AOA communities, of which 46% could be shown on the first two axes (Figure 5A). CCA of the microarray-derived community profiles could also significantly be constrained to pH, clay content, and temperature, but additional incorporation of silt content as a constraining variable, as suggested by BioEnv analysis, gave the highest explanatory power and significance (Figure 5B; CCA, *p* < 0.05, total explained inertia = 48%). Gradients CCA1 = 21%, CCA2 = 12%). Gradients in pH and clay content correlated with the first CCA axes in both CCAs, *albeit* in opposite directions, in both CCAs. Gradients in moisture, temperature, and silt content were equally correlated with the first and second CCA axes.

To identify which of the AOA phylotypes contributed significantly to the community dissimilarities, relative abundance patterns of single OTUs were fitted to the CCA biplots. Significant correlation with the CCA ordinations could be found for OTU 2, 5, and 7 in the clone library-based analysis (Figure 5A) and for OTU 2, 6, 7, and 10 in the microarray-based analysis (Figure 5B). None of the fitted OTUs showed a consistent significant correlation in both CCAs. This result was supported by the lack of significant correlations of relative abundances of OTUs determined by both clone library and microarray analyses with all soil properties. Similarly, a comparison of OTU richness, Shannon diversity and *amoA* copy numbers by Tukey's HSD test resulted in insignificant differences between the four soil groups. Moreover, no correlation of soil properties with *amoA* copy numbers, OTU richness and Shannon diversity was found to be significant on basis of both community analysis methods. Standardized Mantel correlations were performed to test for a relationship between AOA community structure and soil properties, however, no significant correlations were observed.

To determine if there were any interactive effects of fertilization and geothermal heating on OTU richness, OTU diversity (addressed by Shannon's H' index of diversity) and archaeal *amoA* gene copy number, two-way ANOVA analysis was performed. OTU richness, as determined by clone library, was found to be influenced by an interaction between fertilization and geothermal heating, although with no significance (*p* = 0.07).

### AOA phylogeny

**Figure 6:**
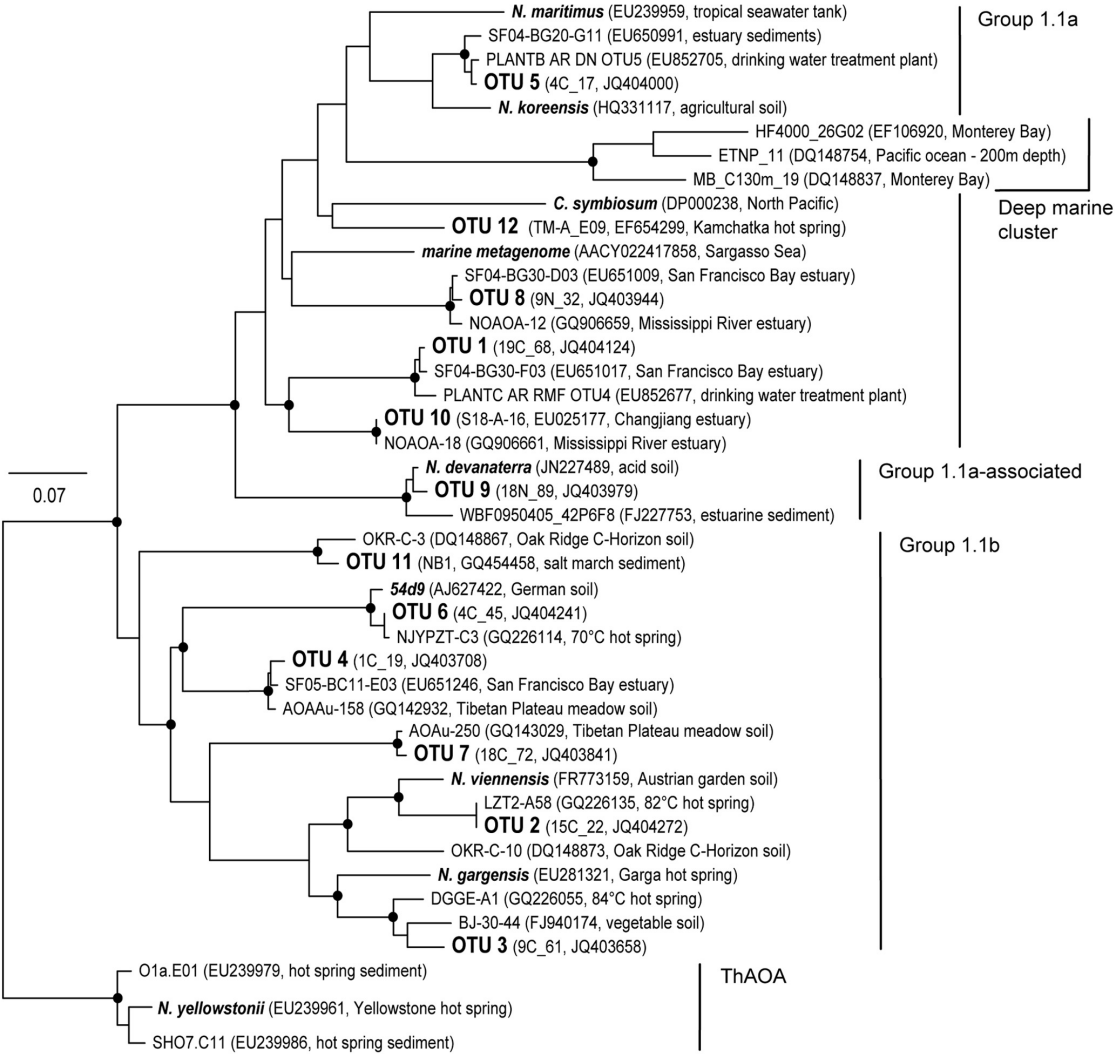
**Neighbor−joining tree based on 1737 *amoA* gene fragments (484 unambiguously aligned positions); only sequences from the work described here as well as selected reference sequences are shown.** Representative *amoA* sequences for phylotype codes (OTUs) defined at 15% nucleotide sequence dissimilarity found in this study are indicated in large font. Phylotype codes are followed by names of representative clones of the *amoA* library and the accession number in brackets. Phylotypes which were only detected by microarray are shown as “Code (name of representative sequence, accession number, isolation source).” Reference sequences are described as “Name (accession number, isolation source).” nodes supported by maximum likelihood method are denoted with closed circles. Scale bar indicates an estimated sequence divergence of 7%. The outgroup is a thermophilic ammonia−oxidizing archaeal lineage containing *Candidatus Nirosocaldus yellowstonii*.

Representative sequences were determined for the 12 AOA *amoA* OTUs and used for the construction of a phylogenetic tree (Figure 6). We found a large phylogenetic diversity among the representative sequences that fell within both major lineages of the AOA tree topology. The sequence representing OTU 1, the most dominant of the 12 OTUs, clustered within group 1.1a and was most closely related to a sequence derived from the San Francisco Bay estuary. Similarly, representative sequences of OTU 4 and 8 were related to sequences obtained from the San Francisco Bay and representative sequences of OTU 10 and 11 had been obtained from estuary sediment and a salt marsh, respectively. Most closely related to the representative sequence of OTU 2, which was the second most dominant phylotype and fell within the *Nitrososphaera viennensis* cluster of the 1.1b lineage, was a sequence retrieved from an 82°C hot spring. Likewise, OTUs 3, 6, and 12 were found to be closely related to sequences representative of hot spring environments. The representative sequence of OTU 9, which was absent in any of the soils analysed in our study with a pH higher than seven, clustered within the 1.1a-associated *Candidatus Nitrosotalea devanaterra* clade.

## Discussion

### Abundance of AOA and AOB

Overall, AOA *amoA* copy numbers were one to three orders of magnitude lower than previously observed in temperate grassland and/or pasture soils (Leininger et al., 2006; Di et al., 2009, 2010; Shen et al., 2011), but in the range of previously described Antarctic soils (Jung et al., 2011). AOB communities were undetectable by quantitative PCR. Although it is quite well established that ratios of archaeal and bacterial *amoA* genes in soil environments can vary greatly (see for example, Leininger et al., 2006; He et al., 2007; Boyle-Yarwood et al., 2008; Shen et al., 2008; Zhang et al., 2009; Di et al., 2010; Adair and Schwartz, 2011; Chen et al., 2011), it is still remarkable to find such low levels of AOB abundance. We expected the bacterial ammonia-oxidizing community to show increased abundance and altered community structure in response to the addition of mineral N fertilizer, as previously reported in soils (Levicnik-Hofferle et al., 2012; Wertz et al., 2012 and reviewed in Schleper and Nicol, 2010). This opposite finding might be explained by the high water-holding capacity and corresponding moisture contents, characteristic of the Andisol classification. Archaeal ammonia oxidizers are likely to be capable of thriving under low oxygen conditions, which are often accompanied by such high water contents, as they are proposed to have lower oxygen demands, due to a different mechanism of ammonia oxidation requiring less oxygen per NH^+^_4_ oxidized (Walker et al., 2010). In fact, AOA have been found in various oxygen-depleted environments (reviewed in Erguder et al., 2009; Park et al., 2010; Schleper and Nicol, 2010), while AOB are generally restricted to more oxic habitats.

We cannot exclude an inhibitory effect of undetermined soil properties on AOB growth and activity. Nevertheless, our results allow us to state that the absence of AOB from soils in cold climates (Nemergut et al., 2008; Alves, 2011) might not be due to temperature, as we were unable to quantify AOB in both geothermally heated and ambient temperature soils. Previous studies reporting (active) AOB populations in cold climate environments additionally reject cold temperature as a general factor inhibiting AOB in soil (Yergeau et al., 2010; Banerjee and Siciliano, 2012; Petersen et al., 2012).

### Structure of AOA communities

Limited effects of geothermal heating and fertilization on distribution and community structure of AOA were observed. This is particularly surprising as temperature, which is known to cause community shifts of AOA (Tourna et al., 2008), varied by at least 8°C between the geothermally heated and ambient temperature soils. Our results show that fertilization may affect AOA diversity irrespective of soil temperature. We found less diverse communities in fertilized soils of both geothermally heated and ambient temperature soils; however, we only observed a decrease of richness in geothermally-heated soils that had been fertilized. The lack of a more pronounced fertilizer effect on the AOA community structure is not remarkable, since previous studies have shown that the application of mineral fertilizer does not alter AOA community structure or size and is of minor importance for AOA in soils (Shen et al., 2011; Levicnik-Hofferle et al., 2012; Wertz et al., 2012). It seems likely that the applied nitrogen within the fertilizer was not available to the ammonia-oxidizing community due to uptake by plants. Indeed, we have evidence of a fertilizer effect on plant biomass and enhanced N uptake by plants at all fertilized sampling sites (Bas Dingemans and Mariet M. Hefting, unpublished results). These observations suggest that the application of mineral N fertilizer could not directly alter microbial communities and that conditions in soils of ambient and increased temperatures were ammonium-limited. None of the soil properties differed between fertilized and non-fertilized soils and we need to assume that the observed changes in diversity and richness of AOA communities in the fertilized soils were caused by not measured factors.

Dissimilarities in community structure of AOA were better explained by the soil properties pH, moisture and clay content and by the temperature gradient rather than by fertilization and geothermal influence as categorical factors. Constrained ordination analysis with both clone library and microarray data supported these relationships. Some of the properties can however not be interpreted as independent factors, since they were correlated with other soil properties; e.g., clay content was negatively correlated with soil moisture content. Therefore, it is not possible to infer absolute magnitudes of pH, moisture, and clay content correlations with AOA community structures. Nevertheless, we found that pH and clay content showed the strongest correlations with AOA communities in all soils analyzed, indicating them to constitute two niche axes of archaeal ammonia oxidizers in soil, as previously proposed for pH (He et al., 2007; Nicol et al., 2008; Zhang et al., 2012), but to our knowledge not for clay content.

### Phylogenetic diversity and response of AOA phylotypes to environmental gradients

The grassland soils studied here harbored a broad phylogenetic diversity of AOA, with a larger fraction of the detected phylotypes clustering within the “marine” 1.1a lineage than usually observed in soils (Pester et al., 2011; Wessen et al., 2011). Furthermore, a substantial number of AOA phylotypes were closely related to sequences from marine, estuarine and hot spring environments, similar to the AOA communities described in Reigstad et al. (2008). These authors sampled hot springs at the same study site. It is reasonable to assume a spreading of these phylotypes by steam (Bonheyo et al., 2005; Ellis et al., 2008) from the abundant hot springs and streams in proximity of the sampling sites into the grassland soils.

We found none of the specific AOA phylotypes to be consistently and significantly correlated with any of the soil physico-chemical gradients. It is possible that the environmental gradients most likely to select for unique AOA phylotypes like pH, clay, and inorganic N contents were too variable within a soil group to allow for statistical separation of community differences. However, our results suggest that only a subset of the AOA phylotypes present in the analyzed soils were adapted to the higher temperatures of the geothermally heated soils. NMS analysis of AOA communities revealed less variability among the geothermally heated soils than among the ambient temperature soils, suggesting a selection for more specific communities in the heated soils. This is supported by AOA community profile analysis showing that geothermally heated soils were only comprised of a subset of the OTUs found in the ambient temperature soils. The “soil” lineage-affiliated OTUs 6 and 7 were mostly absent in the geothermally heated soils. Accordingly, significant increases of “marine” phylotypes, but not of “soil” phylotypes, in response to elevated temperatures have previously been reported (Tourna et al., 2008; Ijichi and Hamasaki, 2011). The absence of OTU 3 from all fertilized soils and the consistent occurrence of OTU 6 in fertilized soils only indicate that some of the AOA phylotypes may be more or less adapted to indirect effects of N fertilization than others in the studied soils.

In line with the observations of Gubry-Rangin et al. (2011), we found the phylotype OTU 9, which was absent in soils with a pH higher than seven, to cluster with the 1.1a-associated *Candidatus Nitrosotalea devanaterra* clade, that is proposed to be adapted to low pH. Therefore, our data suggests that some AOA lineages have a distinct response to temperature, N fertilization and pH, as reviewed in Erguder et al. (2009) and Schleper and Nicol (2010) and recently put forward by Yao et al. (2011) and Szukics et al. (2012), designating these factors to play a role in shaping niches for specific AOA lineages.

## Conclusion

We found substantial dominance of AOA over AOB across a range of *in situ* temperatures in the grassland soils of this study, indicating selection for archaeal ammonia-oxidizers over their bacterial counterpart in these soils independent from temperature. The community structure of AOA was strongly related to pH and clay content, whereas soil temperature and N fertilization played a secondary role. Their primal effect on AOA community structure advocates pH and clay content as universal factors involved in niche-differentiation of AOA in soil.

Even though the specific characteristics of Andisols set our study system apart from most other soil studies on ammonia-oxidizing communities, our findings of AOA dominance and limited response to fertilization and warming support observations in other (Artic) soil environments (Nemergut et al., 2008; Alves, 2011; Lamb et al., 2011; Weedon et al., 2012). A question remaining is what causes such striking absence of AOB. Additionally, the question whether closely related AOA phylotypes perform equally well in different soil habitats (e.g., in respect to temperature) or the AMO protein is conserved in otherwise physiologically distinct AOA, as discussed in Alves (2011), continues to await an answer. Future research, including temporal analysis of *amoA* gene expression, measurements of nitrification rates and confined microcosm experiments, will help to clarify these questions.

### Conflict of interest statement

The authors declare that the research was conducted in the absence of any commercial or financial relationships that could be construed as a potential conflict of interest.

## Appendix

**Table A1:** **PCR primers and cycling conditions applied to amplify AOA**.

**PCR**	**Primer**	**Reaction mixture**	**Cycling conditions**	**Specificity**	**References**
qPCR/	ArchamoA-1F/ArchamoA-2R	12.5 μL ABsolute Q-PCR SYBRgreen mix (AbGene), 1.25 μL of each primer (4 pmol μL^−1^), 10 μg bovine serum albumin (100 mg ml^−1^, New England Biolabs Inc) and 5 μL template DNA (approximately 50 ng total soil DNA); final volume was 25 μl	15 min at 95°C, followed by 40 cycles of 45 s at 95°C, 45 s at 55°C and 45 s at 72°C, and 5 min at 72°C	Archaeal *amoA*	Francis et al., 2005
PCR	ArchamoA-1F/ArchamoA-2R	3 μl sample (~ 50 ng DNA), 2 μl BSA (100 mg ml^−1^, New England Biolabs Inc), 12 μl 10×GoTaq Flexi buffer (Promega), and 1.8 μl MgCl_2_ (25 mM, Promega), 2.50 μl of dNTPs (5 mM, Invitrogen), 2 μl of each primer (5 pmol), and 0.5 μl of GoTaq Flexi polymerase (5 U μl^−1^, Promega); final volume was 50 μl.	5 min at 94°C, followed by 35 cycles of 1 min at 93°C, 1 min at 60°C and 1 min at 72°C, and 5 min at 72°C	Archaeal *amoA*	Francis et al., 2005

**Table A2:** **PCR primers and cycling conditions applied to amplify AOB**.

**PCR**	**Primer**	**Sequence (5′-3′)**	**Cycling conditions^a^**	**Specificity**	**References**
qPCR/PCR	amoA-1F	GGGGTTTCTACTGGTGGT	5 min at 95°C, followed by 45 cycles of 45 s at 95°C, 45 s at 58°C and 45 s at 72°C, and 5 min at 72°C	β-Proteo-bacterial amoA	Rotthauwe et al., 1997
	amoA-2R	CCCCTCKGSAAAGCCTTCTTC			Rotthauwe et al., 1997
qPCR/PCR	amoA-1F	GGGGTTTCTACTGGTGGT	5 min at 95°C, followed by 45 cycles of 45 s at 95°C, 45 s at 58°C and 45 s at 72°C, and 5 min at 72°C	β-Proteo-bacterial amoA	Rotthauwe et al., 1997
	amoA-2R-new	CCCCTC**B**G**S**AAAVCCTTCTTC			Hornek et al., 2006
PCR	amoA-1F	GGGGTTTCTACTGGTGGT	5 min at 95°C, followed by 30 cycles of 30 s at 93°C, 45 s at 57°C and 45 s at 72°C, and 5 min at 72°C	β-Proteo-bacterial amoA	Rotthauwe et al., 1997
	amoA-2R'	CCTCKGSAAAGCCTTCTTC			Okano et al., 2004
1. step PCR	A189	GGNGACTGGGACTTCTGG	3 min at 94°C, followed by 13 cycles of 46 s at 94°C, 90 s at 56°C and 180 s at 72°C, and 10 min at 72°C	β-Proteo-bacterial amoA	Holmes et al., 1995
	amoA-2R	CCCCTCKGSAAAGCCTTCTTC			Rotthauwe et al., 1997
2. step PCR	amoA-1F	GGGGTTTCTACTGGTGGT	3 min at 95°C, followed by 30 cycles of 46 s at 94°C, 30 s at 55°C and 180 s at 72°C, and 10 min at 72°C	β-Proteo-bacterial amoA	Rotthauwe et al., 1997
	amoA-2R	CCCCTCKGSAAAGCCTTCTTC			Rotthauwe et al., 1997
1. step PCR	βAOBf	TGGGGRATAACGCAYCGAAAG	2 min at 95°C, followed by 35 cycles of 30 s at 95°C, 30 s at 59°C and 45 s at 72°C, and 5 min at 72°C	β-Proteo-bacterial 16S rDNA	McCaig et al., 1994
	βAOBr	AGACTCCGATCCGGACTACG			McCaig et al., 1994
2. step PCR	CTO189f	(GC)- GGA GGA AAG CAG GGG ATC G and (GC)- GGA GAA AAG CAG GGG ATC G and (GC)- GGA GGA AAG TAG GGG ATC G	2 min at 95°C, followed by 35 cycles of 30 s at 95°C, 30 s at 59°C and 45 s at 72°C, and 5 min at 72°C	β-Proteo-bacterial 16S rDNA	Kowalchuk et al., 1997
	CTO654r	CTAGC(CT)TTGTAGTTTCAAACGC			Kowalchuk et al., 1997

a Variations on cycling conditions and DNA template concentrations were tested. DNA templates were cleaned with DNA cleanup Wizard (Quiagen). For qPCR ABsolute Q-PCR SYBRgreen mix (AbGene) and SYBR Premix Ex Taq (Takara) and for regular PCR Flexi buffer (Promega) and 2× PCR PreMix F (Epicentre Technologies) were tested.

**Table A3:** **Microarray probes and the accession numbers of the representative sequences for the corresponding phylogenetic clades which were included in the analysis of the microarray data**.

**Probe name**	**Acc number of representative sequence for targeted clade**	**OTU**
AamoA-7	EU671451	6
AamoA-8	EU671451	6
AamoA-9	EU672376	6
AamoA-20	EU239961	2
AamoA-22	EU239961	2
AamoA-26	DQ672634	3
AamoA-41	JF748270	4
AamoA-42	JF748270	4
AamoA-50	GQ454458	11
AamoA-64	GQ142945	7
AamoA-65	GQ142945	7
AamoA-74	GQ143244	7
AamoA-101	GQ142612	9
AamoA-124	EU099951	8
AamoA-132	GQ390323	8
AamoA-133	GQ390323	8
AamoA-134	GQ911228	1
AamoA-135	GQ911228	1
AamoA-136	GQ911228	1
AamoA-137	GU561919	1
AamoA-139	HM160495	1
AamoA-140	HM160495	1
AamoA-142	EU025177	10
AamoA-143	EU025177	10
AamoA-144	FJ543236	5
AamoA-146	EF654299	12
AamoA-147	EF654299	12
AamoA-175	DQ148665	5
AamoA-176	EU651120	5
AamoA-177	EU651120	5
AamoA-178	AB373323	5

Probes with identical representative sequences target the same phylogentic clade and were treated as redundant probes.
